# Leg restlessness and hyperparathyroidism in Parkinson's disease, a further clue to RLS pathogenesis?

**DOI:** 10.3389/fneur.2023.1113913

**Published:** 2023-02-16

**Authors:** Massimo Marano, Valeria Pozzilli, Alessandro Magliozzi, Gaia Tabacco, Anda Mihaela Naciu, Andrea Palermo, Vincenzo Di Lazzaro

**Affiliations:** ^1^Unit of Neurology, Neurophysiology, Neurobiology and Psichiatry, Department of Medicine and Surgery, Università Campus Bio-Medico di Roma, Rome, Italy; ^2^Fondazione Policlinico Universitario Campus Bio-Medico, Rome, Italy; ^3^Unit of Metabolic Bone and Thyroid Disorders, Department of Medicine and Surgery, Università Campus Bio-Medico di Roma, Rome, Italy

**Keywords:** sleep, vitamin D, restless legs, dopamine agonist, parathormone

## Abstract

**Background:**

Non-motor manifestations are the main features of Parkinson's disease (PD). These have been associated with vitamin D abnormalities, but the role of parathormone (PTH) is still obscure. Among the non-motor symptoms of PD, the pathogenesis of restless leg syndrome (RLS) is still debated, but it has been associated with the vitamin D/PTH axis in other disease models. Our study deepens the association between vitamin D and PTH with the prevalence of non-motor symptoms of PD and explores such a relationship in patients reporting leg restlessness.

**Methods:**

Fifty patients with PD were extensively investigated with motor and non-motor scales. Data on serum levels of vitamin D, PTH, and related metabolites were obtained, and patients were stratified as having vitamin D deficiency or hyperparathyroidism according to standardized criteria.

**Results:**

Overall, 80% of patients with PD exhibited low vitamin D levels, and hyperparathyroidism was diagnosed in 45%. The analysis of the non-motor symptoms profile using the non-motor symptom questionnaire (NMSQ) revealed 36% of leg restlessness, a main feature of RLS. This was significantly associated with worse motor symptoms, quality of sleep, and quality of life. Moreover, it was associated with hyperparathyroidism (OR: 3.48) and with PTH levels, independent of vitamin D, calcium/phosphate levels, and motor status.

**Conclusion:**

Our results suggest a significant association between the vitamin D/PTH axis and leg restlessness in PD. PTH has a putative role in nociceptive modulation, and previous evidence on hyperparathyroidism has suggested a possible interrelation with RLS. Further investigations are necessary to add PTH to the non-dopaminergic non-motor landscape of PD.

## Introduction

Parkinson's disease (PD) is characterized by motor and non-motor symptoms. The latter are the main determinants of quality of life and are known to cover several pathophysiological underpinnings of Parkinson's disease, being involved in the prodromal phase and in milestones of the progression through stages (1). In this regard, some of them including leg restlessness, poor quality of sleep, and behavioral disorders are of key importance. Not much is known about the underlying etiologies of such symptoms, and if serum biomarkers may correlate with such manifestations. Patients with PD are also known to exhibit lower 25(OH)D levels than the general population. Indeed, there is growing evidence about the association between vitamin D and non-motor manifestations of PD–cognition, mood, autonomic functions, and falls (2). The presence of insufficient 25(OH)D levels is associated with insomnia, a lower quality of sleep, and a bad disease profile overall (3). Moreover, there are several studies on the role of parathormone (PTH), closely interconnected with vitamin D, in the pathogenesis of restless leg syndrome (RLS) in other disease models, such as end-stage renal disease (4, 5). The link between vitamin D, PTH, and PD patient's non-motor and sleep profiles, however, has not yet been investigated. Both molecules are strictly interconnected. PTH is produced by the parathyroid glands to maintain the right balance of calcium, phosphate, and vitamin D in the bloodstream. Parathyroid levels are controlled by a feedback loop of calcium levels, where low levels of calcium stimulate parathyroid hormone release (6). Hyperparathyroidism may be classified as primary, due to a disorder of the glands, or secondary, thus due to hypocalcemia, frequently secondary to low vitamin D levels (7). The aim of the present study is to investigate the relationship between the vitamin D—PTH axis and non-motor symptoms. Hence, we prospectively studied such associations in a well-structured sample of patients with PD.

## Methods

Patients with PD aged 55–80 years were consecutively enrolled in the outpatient PD clinic of our institution in the winter season of 2020–2021 (21 December to 20 March). We excluded subjects with diseases that could affect bone and calcium metabolism, and administration of drugs affecting calcium concentrations other than peroral vitamin D supplements. All our patients were evaluated by an experienced rater to collect data on disease duration (years), modified Hoehn and Yahr scale (H&Y), Unified Parkinson's Disease Rating scale (UPDRS) part 1 to 4, Non-motor Symptom Questionnaire (NMSQ), Parkinson's Disease Quality of Life Questionnaire (PDQ-39), Montreal Cognitive Assessment (MoCA), and PD Sleep Scale (PDSS). Data on comorbidities, PD therapies, vitamin D supplementations, and dietary oral intake of calcium were also obtained. All the patients were on chronic treatment with levodopa and were tested on their ON-DOPA condition during the morning. Blood samples were collected during the same evaluation to obtain data on 25(OH)D, PTH, calcium, and phosphate levels. Creatinine, blood urea nitrogen (BUN), glomerular filtration rate (GFR), and albumin were also collected to provide corrections to the 25(OH)D and the calcium/phosphate metabolism. The presence of hyperparathyroidism was identified by the PTH cut-off value of >85 pg/ml, while deficient or insufficient 25(OH)D was identified by the cut-off values of <30 and <20 ng/ml, respectively (8). Patients with ongoing 25(OH)D supplementation were included to observe the effect of peroral therapies on the variables investigated. Total serum calcium and serum albumin were measured using automated methods. Serum phosphate and creatinine were also measured by automated techniques. 25(OH)D was measured by an immunochemiluminometric assay (Abbott Laboratories Diagnostics Division, Abbott Park, IL, 60064, USA). Intact PTH was measured by an immunochemiluminometric assay using the automatic analyzer Modular E170 (Roche Diagnostics, Indianapolis, Ind, USA) in the laboratory of our institution. Data were reported as median (QII–QIII) or frequencies (%). Inferential statistics were performed through the Wilcoxon test or the chi-square test according to the distribution. Correlations between variables were tested with Spearman's ρ and the degree of the association with logistic regression or generalized linear modeling. Statistics were performed through the JMP software (SAS, v16.0). The study was conducted according to the Declaration of Helsinki principles and all subjects signed informed consent. The datasets generated during and/or analyzed during the current study are available from the corresponding author upon reasonable request.

## Results

### Clinical, demographic, and biological characteristics of the PD cohort

Our sample included 50 subjects, 17 (34%) were women. The median age was 69.5 (61.7–74) years, and the median disease duration was 6 (3–10) years. The UPDRS part 3, H&Y, and MoCA scores had median values of 20 (15–25), 2 (2–2.5), and 24 (22–26), respectively. The median sleep quality as reported by the PDSS was 94.5 (79.25–109.75). All subjects were on levodopa, with 15 (30%) also taking a dopamine agonist (DA) (total LEDD 600 mgs, 482.5–957.5). Serum levels of 25(OH)D, PTH, calcium, phosphate, and calcium dietary intake are reported in Table 1. Twenty-one (43%) and 15 (35%) patients showed deficient and insufficient 25(OH)D levels, respectively. Hyperparathyroidism was diagnosed in 21 subjects (45%). Twelve patients were on peroral vitamin D supplements (24%). Calcium and phosphate levels were within the normal range, as well as creatinine, BUN, and albumin. All subjects had a GFR >60 ml/min.

**Table 1:** Serum PTH, vitamin D, and related metabolites in our PD cohort.

**Variable [reference range]**	**Median (QI-QIII)**
PTH [14–85 pg/ml]	83.35 (66.08–105.35)
25(OH)D [20–50 ng/ml]	21.7 (15.5–27.7)
Calcium [8.4–10.2 mg/dl]	9.2 (9.2–9.4)
Phosphate [2.3–4.7 mg/dl]	3.1 (2.8–3.43)
Calcium dietary intake (mgs per day)	716.5 (512.5–978.25)

PTH, parathormone.

### Correlations between vitamin D and PTH metabolism with non-motor symptoms and sleep

Patients reported a median of 10 (7–14) non-motor symptoms at NMSQ. There was no association between 25(OH)D, PTH, and sleep as assessed through the PDSS. Vitamin D was significantly lower in patients who gave a positive answer to the NMSQ questions about memory impairment (*p* = 0.036), while PTH levels were higher in patients with constipation (*p* = 0.044), trouble in having sex (*p* = 0.021), and leg restlessness (*p* = 0.020) (Supplementary Table 1). To further verify the clinical significance of such associations, we analyzed the relationship between NMSQ question outcomes and the presence of 25(OH)D insufficiency or deficiency and hyperparathyroidism, according to the established criteria (see Methods section). The only significant association, maintained upon such stratification, was between the NMSQ question 26 (“presence over the last month of unpleasant sensations in legs at night or while resting, and a feeling that they needed to move”) and hyperparathyroidism (64.7 vs. 34.5%, *p* = 0.045). Hence, the sample was consequently stratified accordingly in restless PD (rPD, *n* = 18, 36%) vs. non-restless PD (nrPD).

### Characterization of patients with leg restlessness and correlations with the vitamin D/parathormone axis

The presence of leg restlessness was higher in the female sex; it was associated with higher UPDRS part 3 and PDQ-39 scores and with lower MoCA and PDSS scores (Table 2). Groups (nrPD vs. rPD) did not differ in their LEDD and DA therapy consumption. The rPD group was strongly associated with the PDQ-39 score (ρ 0.670; *p* < 0.001) and with the PDSS score (ρ −0.340; *p* = 0.006). The former association occurred in an independent fashion with respect to sleep quality in a multivariate model. Similarly, the relationship between rPD and quality of life was maintained after correcting for age, sex, and motor status (UPDRS part 3) in a multivariate generalized linear model (Supplementary Table 2). 25(OH)D, calcium, phosphate, dietary calcium, and vitamin D intake were equally distributed across groups. Similarly, creatinine, BUN, and albumin were similar across groups and were excluded for further analysis. As previously mentioned, serum PTH and the prevalence of hyperparathyroidism were higher in the rPD than in the nrPD group (Table 2; unitary odds ratio for PTH pg/ml is 1.02; odds ratio of having RLS in patients with hyperparathyroidism vs. patients without is 3.48).

**Table 2:** Demographic data, disease features, vitamin D (25(OH)D), and PTH-related parameters distributed according to leg restlessness.

**Variables**	**nrPD (*n* = 32)**	**rPD (*n* = 18)**	***p*-value**
**Demographic and disease features**			
Age (years)	69.5 (62.5–73)	70.5 (59.8–76.3)	0.675
Sex (F)	6 (18.8%)	11 (61.1%)	**0.004**
Disease duration (years)	5 (2–10)	7 (3–9.25)	0.905
Modified Hoehn and Yahr score	2 (2–2)	2.5 (1.875–3)	0.088
UPDRS part 3 total score	18 (13.5–21)	25 (19.5–33.25)	**0.010**
MoCA total score	25 (23–26)	23 (21.25–25.25)	**0.026**
LEDD (mgs)	562 (406.5–880)	800 (500–1,095)	0.901
Use of dopamine agonist	8 (25%)	7 (38.8%)	0.347
PDSS	103 (84.5–116.5)	82.5 (61.75–96.25)	**0.005**
PDQ-39	14.21 (7.9–14.2)	44 (32–56)	**< 0.001**
**Metabolic parameters**			
25(OH)D (ng/ml)	22 (14.9–27.44)	20.95 (16.65–30.25)	0.386
25(OH)D deficiency	13 (42%)	8 (47%)	0.806
25(OH)D insufficiency	12 (38.7%)	5 (29.5%)	
Normal 25(OH)D	6 (19.3%)	4 (23.5%)	
PTH (pg/ml)	75.5 (58.6–99.7)	98.8 (69.45–116.65)	**0.020**
Hyperparathyroidism	10 (34.5%)	11 (64.7%)	**0.045**
Vitamin D supplementation	8 (25%)	4 (22%)	1.000
Calcium intake (mgs)	749 (518–979)	681 (496–846)	0.379

UPDRS, Unified Parkinson's Disease Rating Scale; MOCA, Montreal Cognitive Assessment; LEDD, levodopa equivalent daily dose; PDSS, Parkinson's disease sleep scale; PDQ-39, Parkinson's disease questionnaire-39. Statistical significance in bold.

By means of a generalized linear model, there was no effect of sex in the relationship between rPD and PTH (*p* = 0.037). A similar result was observed also after adding the age of the patients in the multivariate model. To investigate the association between PTH, 25(OH)D, calcium, phosphate levels, and calcium dietary intake with rPD, which was selected as a dependent variable, a further model was created. Such analysis confirmed the presence of an independent relationship between PTH levels and leg restlessness (*p* = 0.021), also after adding in the same model the UPDRS part III score as a covariate (*p* = 0.041) (Supplementary Table 3).

Finally, to check the effect of peroral 25(OH)D supplementation, we stratified the sample accordingly and observed that patients with rPD who did not receive 25(OH)D supplements had significantly higher PTH levels and lower PDSS values than nrPD (Table 3).

**Table 3:** Prevalence of leg restlessness in patients with or without 25(OH)D supplementation.

**25(OH)D un-supplemented**	**nrPD (*n* = 22)**	**rPD (*n* = 13)**	***p*-value**
PTH	75.7 (60–104)	98.8 (78.7–118)	**0.031**
25(OH)D	19.3 (14–23.6)	18.5 (16.6–30.2)	0.772
PDSS	106 (81.75–116.5)	87.5 (66–96.5)	**0.021**
**25(OH)D supplemented**	**nrPD (*****n*** = **7)**	**rPD (*****n*** = **4)**	***p*** **-value**
PTH	75 (42–87.5)	84.5 (60–118)	0.780
25(OH)D	33.4 (27.8–43.7)	22.5 (16–30)	**0.018**
PDSS	93 (86.25–118)	71 (59.25–92.75)	0.174

PTH, parathormone; PDSS, Parkinson's disease sleep scale. Statistical significance in bold.

## Discussion

Vitamin D and its hormonal axis are involved in Parkinson's non-motor profile. This is further confirmed by the present study, which is in line with the available literature and with its heterogeneity (3). The latter is probably caused by differences in the demographic sample characteristics, in the outcome measures, and by the biological variability of the 25(OH)D and PTH metabolism over time, during seasons due to light exposure, but also age and sex (9). Our real-life study was conducted on a mild-to-moderate sample of patients with PD during the winter season when vitamin D levels are putative to be lower with a possible increase in PTH than in other periods of the year. Nevertheless, the high prevalence of hyperparathyroidism in PD (45% of our cohort) has never been systematically reported. Our study also documented that almost 80% of patients had impaired 25(OH)D levels, with 40% of them bearing insufficient levels (<10 ng/ml). In light of such data, it is not surprising that patients with PD exhibit high PTH levels. In a few anecdotal reports, authors described patients with concomitant parkinsonism and hyperparathyroidism (10, 11), questioning if the latter was an incidental finding or a causative condition; noteworthy surgical removal of the parathyroid glands improved symptoms.

Despite the non-motor symptom screening with NMSQ identified various possible associations with 25(OH)D and PTH (i.e., memory performances, constipation, sexual function, and restless legs), only the link between PTH levels and restless legs maintained after selecting clinically relevant measures of interest (i.e., the presence of insufficiency or deficiency of vitamin D and hyperparathyroidism according to standardized criteria). In line with previous studies obtained with NMSQ (12), we found that 30–40% of patients with PD reported leg restlessness as “unpleasant sensations in legs at night or while resting, and a feeling that they needed to move.” The latter was confirmed to be a strong determinant of sleep quality and quality of life, independent of motor status.

Leg restlessness is a frequent symptom in PD and the epidemiological link between PD, leg restlessness and RLS is complex, as the prevalence of RLS in PD shows diverging results ranging from 0 to 50% (13), with prospective studies identifying a more trustable prevalence of 10–20% (14). Such variability is mainly caused by the heterogeneity of methods used for RLS screening: from having the symptom of “irresistible desire to move the legs, particularly at night” used in the former data on prevalence (15), to the use of the IRLSSG diagnostic criteria (which have undergone two revisions since their first publication in 1995) in the latter (16). Of interest, in our series, patients presented with a median of 10 non-motor symptoms but only the presence of leg restlessness had a direct correlation with higher PTH values and prevalence of hyperparathyroidism.

In this regard, the association between PTH and RLS is not entirely new. High levels of PTH are associated with bad quality of sleep and RLS in patients affected by the end-stage renal disease (4), and even in this case, surgical removal of the parathyroid glands appears to improve symptoms of RLS, hypothesizing that an imbalance between calcium and phosphate levels could be the underlying etiology of the irresistible urge to move the legs (17). Some studies, however, reported no correlation between RLS and biochemical abnormalities including electrolyte levels in patients undergoing hemodialysis (18). In line with this, our study showed no signs of renal impairment or alteration in calcium and phosphate homeostasis. As a result, despite several studies addressing this phenomenon, the association between hyperparathyroidism and RLS is still poorly understood.

Hypothesis on the presence of RLS in patients with PD includes a progressive depletion of the dopaminergic system due to long disease duration or a consequence of long-term antiparkinsonian therapy (19). The sensation of leg motor restlessness (LMR), yet not fulfilling the diagnostic criteria of RLS, has been found to be present also in drug-naive patients with early Parkinson's disease. Such symptoms, however, did not have diurnal fluctuations, reflecting possible akathisia or other causes of restlessness (20). It has been postulated that LMR may be a prodrome of the future development of RLS (21). In our study, patients with PD were all on treatment, and despite the disease duration being similar between groups, patients with rPD had higher motor and lower cognitive scores at UPDRS part 3 and MoCA, respectively. However, the importance of the management of leg restlessness in our patients was supported by the prominent relationship between RLS and quality of life (i.e., PDQ-39), which occurs independently of any other sleep disturbances as evaluated by the PDSS.

The link between hyperparathyroidism and RLS in PD is possible and represents a new therapeutic chance. In our study, the relationship between PTH and leg restlessness appears to be independent of potential confounders—including motor status or age and sex which are known to be involved in PTH dynamics (19). The central nervous system exhibits the parathyroid hormone receptor 2, which is concentrated in the endocrine and limbic regions in the forebrain. Its endogenous ligand, TIP39, modulates several aspects of the stress response, in particular, the nociceptive processing (i.e., facilitating the nociceptive transmission at a supraspinal level), through what is called the neuroendocrine system (22). Accordingly, growing evidence supports the view of RLS as a derangement of sensorimotor interaction and of the gating of nociceptive information to the central nervous system (23) where high PTH levels might have an effect. PTH has been shown to modulate dopamine turnover in the rat *in vivo*, implicating a link between the concentration of two molecules, (24) and their possible interrelation in the pathophysiology of RLS. Furthermore, the two molecules both inhibit phosphate transport in cultured mouse proximal tubule cells, contributing to shared mechanisms in the feedback loop between calcium/phosphate/PTH (25). Elevated parathyroid hormone levels are also associated with poor sleep quality, and parathyroidectomy has been found to improve insomnia substantially (26). Vitamin D has negative feedback on PTH exertion and may therefore be a possible actor in the management of leg restlessness. To corroborate a possible exclusive association between PTH and RLS and in the absence of a more specific scale, it is worthy to report that in our cohort neither NMSQ question 10 (“unexplained pains”) nor UPDRS II question 17 (“sensory complaints related to parkinsonism”) reported a statistical association with PTH (data not shown). Our data, therefore, provide early possible evidence of an effect of 25(OH)D supplementation on PTH and RLS symptoms. The increase in vitamin D levels would play a role in calcium absorption and, consequently, in PTH reduction through a negative feedback loop (27).

The present study has the main limitation of relying on the NMSQ to identify the symptom of leg restlessness and not RLS ascertained through the IRLSSG criteria. The former has a good sensibility (~85%) but a lower specificity (15), owing probably to the fact that it does not account for relief induced by movement. Our sample might contain RLS mimics, such as polyneuropathy and akathisia. Signs or symptoms of such conditions were not reported in the clinical routine of our cohort, but given that the protocol was not designed to address such conditions, further studies are warranted to verify our hypothesis. In our opinion, however, our results deserve to be shared to allow replication studies with a more complete methodology (e.g., RLS criteria and rating scales, bone metabolism instrumental investigation, neurophysiological tests) on larger controlled samples. Moreover, the lack of consistency in the link between PTH and restless legs vs. PTH and pain questions at NMSQ or UPDRS II reinforces our hypothesis. Prospective longitudinal data would be of further help in characterizing the associations between PTH and PD. Despite our preliminary results being compatible with the presence of a possible effect of 25(OH)D supplementation on PTH and RLS symptoms, a study with a specific design is furtherly warranted. We may speculate, therefore, that in predisposed individuals, such as patients with PD, PTH may preferentially act as a neuromodulator able to enhance non-motor symptoms such as leg restlessness, probably through a non-dopaminergic pathway.

**Figure 1:**
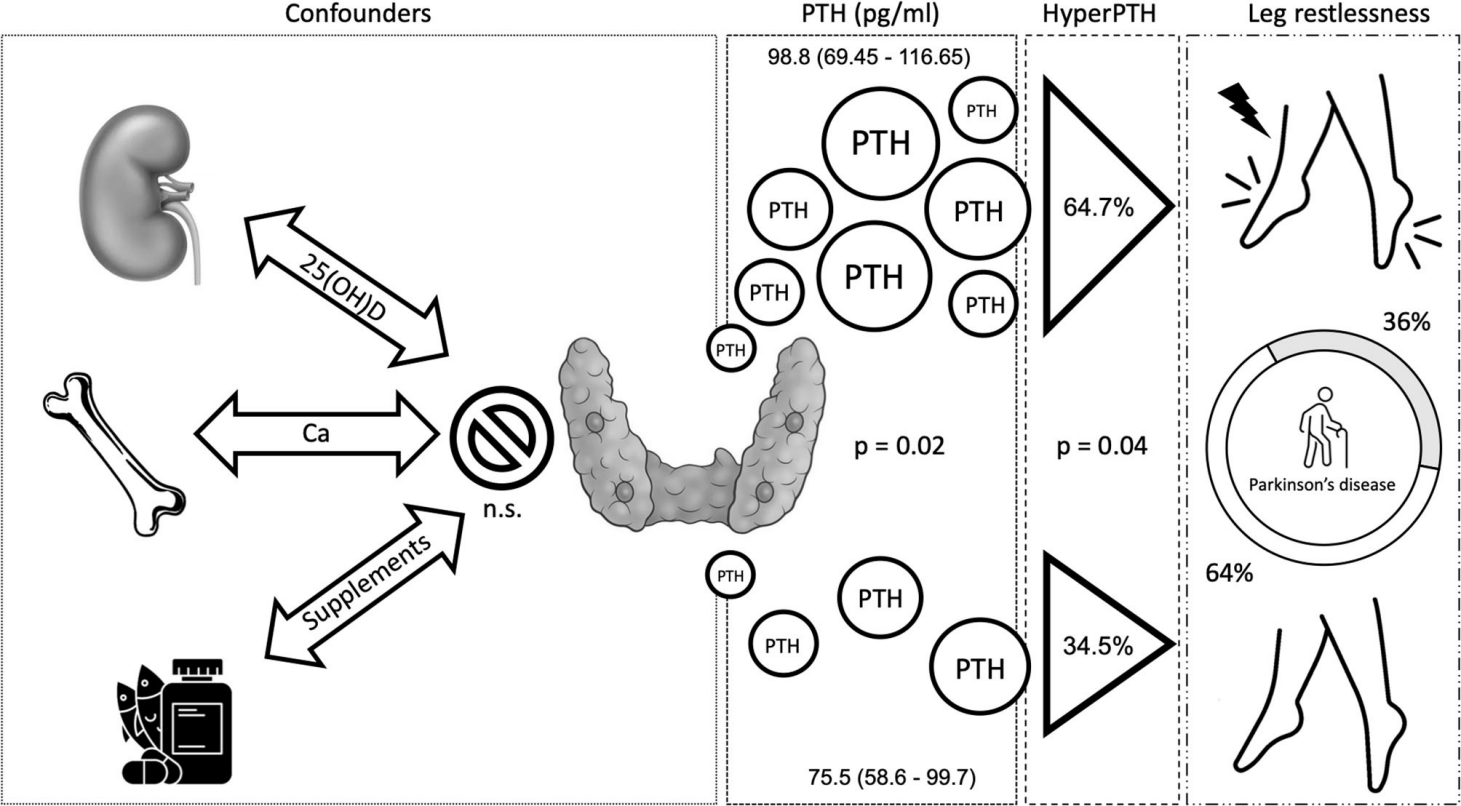
Hypothetical interrelation between hyperparathyroidism and leg restlessness.

In conclusion, PTH, but not calcium, phosphate, or even vitamin D itself, is associated with the presence of RLS symptoms in PD, and such relationship is not significantly influenced by the patient's motor features (Figure 1). Leg restlessness may, indeed, be improved using vitamin D, allowing us to hypothesize future pathophysiologic and therapeutic scenarios for leg restlessness in patients with PD.

## Data availability statement

The raw data supporting the conclusions of this article will be made available by the authors, without undue reservation.

## Ethics statement

The studies involving human participants were reviewed and approved by Ethical Committee Campus Bio-Medico University, Rome. The patients/participants provided their written informed consent to participate in this study.

## Author contributions

MM: data collection, study design, first draft writing, and statistical analysis. VP: first draft writing. AM: data collection and study design. AP: study design. VD, GT, VP, AP, and AN: review and critique. All authors contributed to the article and approved the submitted version.
