# Supplementary material for: Leg restlessness and hyperparathyroidism in Parkinson's disease, a further clue to RLS pathogenesis?
Source: Front Neurol. 2023 Feb 16;14:1113913. doi: 10.3389/fneur.2023.1113913 (PMC9978794; doi:10.3389/fneur.2023.1113913)
Supplement: Supplementary file 1 [file Table_1.docx]

Supplementary Material

Leg restlessness and hyperparathyroidism in Parkinson’s disease, a further clue to RLS pathogenesis?

**Massimo Marano, MD, PhD^1,3^; Valeria Pozzilli, MD^1,3^; Alessandro Magliozzi, MD^1,3^; Gaia Tabacco, MD^2^; Anda Mihaela Naciu, MD, PhD^2,3^; Andrea Palermo, MD, PhD^2,3^; Vincenzo Di Lazzaro, MD^1,3^**

^1^Unit of Neurology, Neurophysiology, Neurobiology and Psichiatry, Department of Medicine and Surgery, Università Campus Bio-Medico di Roma, Via Alvaro del Portillo, 21 - 00128 Rome, Italy

^2^Department of Medicine and Surgery, Unit of Metabolic bone and thyroid disorders; Fondazione Policlinico Universitario Campus Bio-Medico, Rome, Italy

**^3^**Fondazione Policlinico Universitario Campus Bio-Medico, Via Alvaro del Portillo, 200 - 00128 Roma, Italy.

**Correspondence:** Massimo Marano, m.marano@policlinicocampus.it

**Supplementary table 1.** Distribution of vitamin D and PTH levels across non-motor symptoms (NSMQ items).

| **Impaired NMSQ** |  | **Vitamin D**  **Median (QI-QIII)** | **Significance (p-value)** | **PTH** | **Significance (p-value)** |
| --- | --- | --- | --- | --- | --- |
| Dribbling of saliva |  |  |  |  |  |
|  | No (n=38) | 22.5 (12.2-28.5) | 0.085 | 75.2 (60.4-105.3) | 0.819 |
|  | Yes (n=12) | 17.35 (15.8-26.8) |  | 90.4 (80.8-116.7) |  |
| Loss or change in taste or smell |  |  |  |  |  |
|  | No (n=38) | 21.7 (16-28.5) | 0.214 | 81.4 (64.5-101.4) | 0.826 |
|  | Yes (n=12) | 20.6 (11.2-27.6) |  | 101 (67.7 – 118.7) |  |
| Difficulty in swallowing |  |  |  |  |  |
|  | No (n=29) | 22 (15.3-28.8) | 0.573 | 85.8 (68.2-122-5) | 0.161 |
|  | Yes (n=21) | 20.9) (15.6-27.4 |  | 75.5 (63.5-98.8) |  |
| Nausea or vomiting |  |  |  |  |  |
|  | No (n=41) | 20.9 (15.3-27.5) | 0.779 | 82.6 (64-190.5) | 0.421 |
|  | Yes (n=9) | 24 (16-34) |  | 84 (66.2-104.3) |  |
| Constipation |  |  |  |  |  |
|  | No (n=28) | 22 (15-25) | 0.699 | 84.8 (70.7-109.9) | **0.044** |
|  | Yes (n=22) | 20.9 (16.3-43-5) |  | 74.9 (50-101-1) |  |
| Bowel incontinence |  |  |  |  |  |
|  | No (n=50) | 21.7 (15.5-27-7) | - | 83.3 (66-105.3) | - |
|  | Yes (n=0) | - |  | - |  |
| Incomplete bowel emptying |  |  |  |  |  |
|  | No (n=28) | 22.5 (15-30.8) | 0.523 | 76 (65.8-101.9) | 0.801 |
|  | Yes (n=22) | 20 (15.9-27.6) |  | 95 (64.4-116-2) |  |
| Urinary urgency |  |  |  |  |  |
|  | No (n=14) | 22.7 (19.3-31.5) | 0.164 | 76 (66.3-95) | 0.728 |
|  | Yes (n=36) | 19.6 (15-39) |  | 84.8 (65.4-109.5) |  |
| Nocturia |  |  |  |  |  |
|  | No (n=11) | 22.6 (20-60.9) | 0.148 | 75.5 (68.3-86.5) | 0.784 |
|  | Yes (n=39) | 20.3 (15-36.8) |  | 85.8 (60.9-107.8) |  |
| Unexplained pains |  |  |  |  |  |
|  | No (n=36) | 21.7 (14.8-28.5) | 0.814 | 80.2 (63.5-105) | 0.757 |
|  | Yes (n=14) | 22 (16.9-28.6) |  | 98.8 (66.7-107.3) |  |
| Unexplained change in weight |  |  |  |  |  |
|  | No (n=47) | 21.4 (15.7-27.4) | 0.600 | 82.6 (65.4-105.1) | 0.810 |
|  | Yes (n=3) | 27.8 (14.4-32-2) |  | 103.5 (74.9 – 122.9) |  |
| Problems in remembering things |  |  |  |  |  |
|  | No (n=31) | 22.5 (15.9-28.5) | **0.036** | 75.75 (62.6 – 94.5) | 0.926 |
|  | Yes (n=19) | 19 (13.5-26) |  | 101.1 (66.8 – 113.5) |  |
| Loss of interest |  |  |  |  |  |
|  | No (n=37) | 21.4 (15.5-27.6) | 0.422 | 83.4 (66.5-104.8) | 0.542 |
|  | Yes (n=13) | 22 (15.2-28.8) |  | 84.5 (64.5-109.7) |  |
| Hallucinations |  |  |  |  |  |
|  | No (n=45) | 20.9 (15.2-27.3) | 0.886 | 84 (66.2-105.6) | 0.362 |
|  | Yes (n=5) | 28.8 (20.5-34.2) |  | 72.6 (61.5-106.9) |  |
| Difficulty concentrating |  |  |  |  |  |
|  | No (n=25) | 24.5 (14.3-32.7) | 0.067 | 75.75 (53.7-105.3) | 0.758 |
|  | Yes (n=25) | 19.9 (15.7 – 24.2) |  | 85.3 (66.5-108) |  |
| Sadness |  |  |  |  |  |
|  | No (n=24) | 21.1 (14.3 – 26.8) | 0.594 | 75.75 (65.1-122.5) | 0.353 |
|  | Yes (n=26) | 22 (15.5 – 30.3) |  | 84.45 (65.8-102.7) |  |
| Anxiety |  |  |  |  |  |
|  | No (n=33) | 22 (14.9-27.6) | 0.357 | 74.9 (61.7-105.1) | 0.844 |
|  | Yes (n=17) | 21.4 (15.8 – 28.8) |  | 92.9 (76-110.4) |  |
| Loss of sex drive |  |  |  |  |  |
|  | No (n=26) | 20.9 (14.6-26.2) | 0.574 | 76 (67-110.4) | 0.339 |
|  | Yes (n=24) | 22.6 (16-30.8) |  | 85.8 (56.5-103.5) |  |
| Sex difficulties |  |  |  |  |  |
|  | No (n=37) | 20.9 (15.8-25) | 0.527 | 85.8 (68.3 – 116.2) | **0.021** |
|  | Yes (n=13) | 25.2 (15.2 – 31.2) |  | 80 (39.3-101.1) |  |
| Orthostasis |  |  |  |  |  |
|  | No (n=24) | 21.7 (15.5-28.2) | 0.226 | 81.4 (86.1 – 106.2) | 0.669 |
|  | Yes (n=26) | 21.7 (15.3-28) |  | 86.1 (66-106.2) |  |
| Falling |  |  |  |  |  |
|  | No (n=34) | 20.6 (15.2 – 26.7) | 0.750 | 75.75 (60.1-109.7) | 0.653 |
|  | Yes (n=16) | 24.7 (15.8 – 31.8) |  | 85.7 (73-104) |  |
| Excessive daytime sleepiness |  |  |  |  |  |
|  | No (n=44) | 22.25 (15.2-29.3) | 0.051 | 82.15 (63 – 104.8) | 0.775 |
|  | Yes (n=6) | 18.5 (16.7 – 24.3) |  | 90.7 (66 – 120.1) |  |
| Sleep problems |  |  |  |  |  |
|  | No (n=22) | 20.3 (15 – 27.5) | 0.787 | 75.5 (64-103.7) | 0.805 |
|  | Yes (n=28) | 22 (16 – 30.8) |  | 84 (66.2-116.2) |  |
| Vivid dreams |  |  |  |  |  |
|  | No (n=25) | 22.5 (17.4 – 27.8) | 0.209 | 76 (63.55-99) | 0.931 |
|  | Yes (n=25) | 19.6 (14.5 – 27) |  | 95 (67-116.6) |  |
| Dream enacting behavior |  |  |  |  |  |
|  | No (n=22) | 24 (19.3 – 31.2) | 0.059 | 75.2 (65.6-91.5) | 0.630 |
|  | Yes (n=28) | 17.5 (14.5 – 25.2) |  | 96.9 (64.4 – 106.9) |  |
| Restless legs |  |  |  |  |  |
|  | No (n=32) | 22 (15-27.5) | 0.386 | 75.5 (58.6-99.7) | **0.020** |
|  | Yes (n=18) | 20.9 (16.6-30.2) |  | 98.8 (69.5 – 98.8) |  |
| Swollen legs |  |  |  |  |  |
|  | No (n=42) | 20.9 (15 – 26.2) | 0.893 | 84.1 (61.7-105.1) | 0.683 |
|  | Yes (n=8) | 27.8 (18.5-36.2) |  | 74.9 (71.6-110.5) |  |
| Excessive sweating |  |  |  |  |  |
|  | No (n=39) | 20.6 (15-27.3) | 0.894 | 84.1 (66.5 – 105) | 0.376 |
|  | Yes (n=11) | 23.9 (17.7-34.9) |  | 74.9 (49.2 – 114) |  |
| Double vision |  |  |  |  |  |
|  | No (n=41) | 20.9 (15.3-27.8) | 0.504 | 82.1 (60.4 – 104.1) | 0.808 |
|  | Yes (n=9) | 24 (15.5-27) |  | 93.3 (67.7 – 109) |  |
| Illusions |  |  |  |  |  |
|  | No (n=47) | 21.4 (15.2 – 27.6) | 0.553 | 82.6 (66.3 – 104) | 0.789 |
|  | Yes (n=3) | 24 (18.5-28.8) |  | 110.4 (65.4 – 151.9) |  |

NMSQ, non-motor symptoms questionnaire. Statistical significance in bold.
