# Supplementary material for: Leg restlessness and hyperparathyroidism in Parkinson's disease, a further clue to RLS pathogenesis?
Source: Front Neurol. 2023 Feb 16;14:1113913. doi: 10.3389/fneur.2023.1113913 (PMC9978794; doi:10.3389/fneur.2023.1113913)
Supplement: Supplementary file 2 [file Table_2.docx]

Supplementary Material

Leg restlessness and hyperparathyroidism in Parkinson’s disease, a further clue to RLS pathogenesis?

**Massimo Marano, MD, PhD^1,3^; Valeria Pozzilli, MD^1,3^; Alessandro Magliozzi, MD^1,3^; Gaia Tabacco, MD^2^; Anda Mihaela Naciu, MD, PhD^2,3^; Andrea Palermo, MD, PhD^2,3^; Vincenzo Di Lazzaro, MD^1,3^**

^1^Unit of Neurology, Neurophysiology, Neurobiology and Psichiatry, Department of Medicine and Surgery, Università Campus Bio-Medico di Roma, Via Alvaro del Portillo, 21 - 00128 Rome, Italy

^2^Department of Medicine and Surgery, Unit of Metabolic bone and thyroid disorders; Fondazione Policlinico Universitario Campus Bio-Medico, Rome, Italy

**^3^**Fondazione Policlinico Universitario Campus Bio-Medico, Via Alvaro del Portillo, 200 - 00128 Roma, Italy.

**Correspondence:** Massimo Marano, m.marano@policlinicocampus.it

**Supplementary table 2.** Multivariated GLM of the association between rPD, quality of life, age, sex and motor status.

| **Variable** | **Estimates** | **Standard error** | **Chi-squared** | **p-value** | **Lower CL** | **Higher CL** |
| --- | --- | --- | --- | --- | --- | --- |
| Intercept | 1.053 | 2.954 | 0.129 | 0.718 | -4.636 | 7.461 |
| PDQ-39 | -0.103 | 0.037 | 12.235 | 0.000 | -0.193 | -0.040 |
| Age | 0.040 | 0.047 | 0.722 | 0.395 | -0.056 | 0.138 |
| Sex (F) | -0.329 | 0.449 | 0.530 | 0.466 | -1.215 | 0.583 |
| UPDRS part 3 | -0.021 | 0.055 | 0.156 | 0.692 | -0.137 | 0.087 |

GLM, generalized linear model; PDQ-39, Parkinson’s disease questionnaire-39; UPDRS, Unified Parkinson's Disease Rating Scale.
