# Supplementary material for: Leg restlessness and hyperparathyroidism in Parkinson's disease, a further clue to RLS pathogenesis?
Source: Front Neurol. 2023 Feb 16;14:1113913. doi: 10.3389/fneur.2023.1113913 (PMC9978794; doi:10.3389/fneur.2023.1113913)
Supplement: Supplementary file 3 [file Table_3.docx]

Supplementary Material

Leg restlessness and hyperparathyroidism in Parkinson’s disease, a further clue to RLS pathogenesis?

**Massimo Marano, MD, PhD^1,3^; Valeria Pozzilli, MD^1,3^; Alessandro Magliozzi, MD^1,3^; Gaia Tabacco, MD^2^; Anda Mihaela Naciu, MD, PhD^2,3^; Andrea Palermo, MD, PhD^2,3^; Vincenzo Di Lazzaro, MD^1,3^**

^1^Unit of Neurology, Neurophysiology, Neurobiology and Psichiatry, Department of Medicine and Surgery, Università Campus Bio-Medico di Roma, Via Alvaro del Portillo, 21 - 00128 Rome, Italy

^2^Department of Medicine and Surgery, Unit of Metabolic bone and thyroid disorders; Fondazione Policlinico Universitario Campus Bio-Medico, Rome, Italy

**^3^**Fondazione Policlinico Universitario Campus Bio-Medico, Via Alvaro del Portillo, 200 - 00128 Roma, Italy.

**Correspondence:** Massimo Marano, m.marano@policlinicocampus.it

**Supplementary table 3.** Multivariated GLM of the association between rPD, PTH, vitamin D and related parameters, and motor status.

| **Variable** | **Estimates** | **Standard error** | **Chi-squared** | **p-value** | **Lower CL** | **Higher CL** |
| --- | --- | --- | --- | --- | --- | --- |
| Intercept | 38.892 | 20.555 | 5.946 | 0.014 | 4.784 | 85.821 |
| PTH | -0.029 | 0.015 | 4.048 | 0.044 | -0.064 | 0.000 |
| Vitamin D | 0.007 | 0.042 | 0.031 | 0.859 | -0.072 | 0.102 |
| Calcium Intake | 0.001 | 0.001 | 1.018 | 0.313 | -0.001 | 0.004 |
| Calcium | -3.227 | -3.227 | 1,971 | 0.059 | -7,685 | 0,079 |
| Phosphate | -1.236 | -1.236 | 1,131 | 0.252 | -3,753 | 0,842 |
| UPDRS III | -0.147 | -0,147 | 0,058 | 0.001 | -0,282 | -0,049 |

GLM, generalized linear model; UPDRS, Unified Parkinson's Disease Rating Scale;
